# Comparative study on the selection of drainage methods in posterior lumbar interbody fusion

**DOI:** 10.1186/s12893-023-02106-3

**Published:** 2023-07-21

**Authors:** Chaoran Wang, Xuewei Wang, Zongqiang Yang, Jiandang Shi, Ningkui Niu

**Affiliations:** 1 Department of Orthopedics, General Hospital of Ningxia Medical University, 804 Shengli South Street, Xingqing District, Yinchuan, 750004 China; 2 School of Clinical Medicine, Ningxia Medical University, Yinchuan, China; 3 Medical Record Room, General Hospital of Ningxia Medical University, Yinchuan, China

**Keywords:** Posterior lumbar interbody fusion, Incision drainage, Natural pressure drainage, Negative pressure drainage

## Abstract

**Objective:**

To compare and analyze the clinical effects of bilateral natural pressure drainage and negative pressure drainage after posterior lumbar interbody fusion (PLIF) to provide a reference for selecting drainage methods after lumbar surgery.

**Methods:**

A retrospective cohort study, 281 patients who underwent single-segment PLIF in our hospital from January 2017 to December 2020 and met the inclusion and exclusion criteria were included in the study, including 132 males and 149 females, aged 22–85 years, with an average of (53.62 ± 11.23) years. According to different postoperative incision drainage methods determined by the random number table method before surgery, they were divided into the natural pressure drainage group and negative pressure drainage group, both of which were bilateral drainage. The general observation indexes and perioperative-related indexes were recorded and analyzed.

**Results:**

There were 143 cases in the natural pressure drainage group and 138 cases in the negative pressure drainage group. There was no significant difference in age, gender, body mass index, disease type, blood pressure on the day of surgery, preoperative albumin, hemoglobin, platelet, prothrombin time, and intraoperative bleeding between the two groups (*P* > 0.05). The albumin on the first postoperative day in the natural pressure drainage group was higher than that in the negative pressure drainage group [(33.24 ± 3.52) vs. (32.17 ± 5.03), *P* < 0.05]; The hemoglobin on the first postoperative day in the natural pressure drainage group was higher than that in the negative pressure drainage group [(126.01 ± 15.03) vs. (115.19 ± 16.25), *P* < 0.01]; The drainage volume on the first postoperative day in the natural pressure drainage group was lower than that in the negative pressure drainage group [(93.25 ± 63.58) ml vs. (119.46 ± 54.48) ml, *P* < 0.01]; The total postoperative drainage volume in the natural pressure drainage group was lower than that in the negative pressure drainage group [(355.60 ± 189.69) ml vs. (434.37 ± 149.12) ml, *P < *0.01]; The indwelling time of drainage tube in the natural pressure drainage group was lower than that in the negative pressure drainage group [(3.29 ± 1.17) d vs. (3.45 ± 0.97) d, *P* < 0.05]. There was no significant difference in platelet count on the first postoperative day, postoperative hospital stays, and complications (incision infection and hematoma) between the two groups (*P* > 0.05).

**Conclusion:**

Bilateral natural pressure drainage and negative pressure drainage can achieve good drainage effects after PLIF, but patients with natural pressure drainage have less loss of albumin and hemoglobin, less drainage volume, and shorter drainage tube indwelling time, which is worthy of clinical application.

## Background

Drainage tube placement in the spinal decompression area is routine to ensure smooth postoperative drainage and reduce hematoma compression and infection [1–4]. With the continuous advancement of minimally invasive concepts and hemostasis technology, there is no unified standard for the number, method, indwelling time, and removal criteria of drainage tubes placed in the operation area. Clinicians usually place drainage tubes according to their experience, including unilateral drainage, bilateral drainage, natural pressure drainage, and negative pressure drainage. There are also options not to put a drainage tube. How to choose a more reasonable, effective drainage method with fewer complications is worth thinking about. Posterior lumbar interbody fusion (PLIF) is a classic operation of posterior lumbar decompression, fixation, and fusion, it is still the operation method commonly used by spine surgeons [5], and there is no unified standard for the placement of drainage tubes. Therefore, this paper compares and analyzes the clinical effects of bilateral natural pressure drainage and negative pressure drainage after single-segment PLIF to provide a reference for the drainage tube placement in the surgical area after PLIF.

## Materials and methods

### Inclusion and exclusion criteria

Inclusion criteria: (1) Confirmed by X-ray, CT, MRI, and other imaging examinations as degenerative and isthmic spondylolisthesis, lumbar stenosis, lumbar disc herniation instability, single-segment intervertebral instability, and degenerative scoliosis; (2) The clinical symptoms and signs of the patients consistent with the clinical manifestations of the corresponding diseases, and the course of the disease lasts for more than 6 months with a decrease in quality of life; (3) Progressive worsening or no significant relief of symptoms after more than 6 months of strict conservative treatment; (4) Implemented single-segment PLIF; (5) Age range from 18 to 85 years old, regardless of gender; (6) Obtained complete follow-up.

Exclusion criteria: (1) Previous history of lumbar spine surgery; (2) Combined lumbar infection, tumor, and deformity; (3) Combined diabetes and severe cardiovascular disease; (4) Preoperative evaluation showed abnormal coagulation function or received blood transfusion treatment during or after surgery; (5) Intraoperative dural rupture or postoperative cerebrospinal fluid leak.

### General information

A retrospective cohort study, 281 patients who underwent single-segment PLIF in our hospital from January 2017 to December 2020 and met the inclusion and exclusion criteria were included in the study, including 132 males and 149 females, aged 22–85 years, with an average of (53.62 ± 11.23) years. Before surgery, patients were assigned to the natural pressure drainage group and negative pressure drainage group using the random table method according to their case IDs. All patients were informed of the drainage method to be used before surgery and written consent was obtained from all patients. According to the different drainage methods, they were divided into 143 cases in the natural pressure drainage group and 138 cases in the negative pressure drainage group.

### Clinical characteristics and evaluation indicators

After hospitalization, patients’ height and weight were measured, body mass index (BMI) was calculated, serum albumin, hemoglobin, platelets, and prothrombin time were measured 24 h before and after surgery. Patients’ blood pressure was measured on the day of surgery, intraoperative blood loss, drainage volume within 24 h after surgery, drainage tube indwelling time, total drainage volume, and postoperative hospitalization stays were recorded. After surgery, closely observe the patients’ vital signs, lower limb nerve function, and incision dressing.

Diagnostic criteria for deep incision infection: (1) Finding purulent fluid through deep incision drainage; (2) Body temperature exceeding 38 ℃, local tenderness or spontaneous pain, or spontaneous dehiscence of the deep incisions; (3) Abscess or other evidence of deep incision infection was confirmed through incision examination, secondary surgery, histopathological diagnosis, and imaging examination; (4) The surgeon made a diagnosis of deep incision infection, or obtained positive results by performing debridement and pus microbiological culture.

Diagnostic criteria for symptomatic hematoma: The possibility of symptomatic hematoma is suspected when the following conditions occur after surgery. (1) Nerve root injury, unilateral or bilateral sciatica; (2) Cauda equina injury, delayed paralysis below the injury level, with sensory and motor dysfunction, loss of sphincter function, decreased muscle tension, disappearance of tendon reflexes, no pathological pyramidal signs; (3) Progressive aggravation of wound and surrounding pain, accompanied by massive wound bleeding or swelling of the wound area. Subsequently, blood routine examination and coagulation function tests were repeated to determine the existence of thrombocytopenia and coagulation dysfunction, and the final diagnosis was confirmed through MRI or surgery.

### Surgical methods

The same medical team operated on all patients. In the prone position, a median longitudinal incision, 4-6 cm long, exposes the spinous process, lamina, facet joint, and the root of the transverse process layer by layer. Four pedicle screws were placed using the Magerl method. The lower 2/3 of the upper lamina of the diseased segment and the upper 1/3 of the lower lamina were removed, the hypertrophic ligamentum flavum was gradually removed, and the lateral recess was enlarged. Decompression until bilateral nerve roots are released without compression. The intervertebral disc was scraped clean and filled with autologous bone, an intervertebral cage was placed, and compression fixation between the vertebral bodies was finally performed. Irrigated the incision using normal saline 1500 ml, and posterolateral autogenous and allogenic bone grafts were performed. Hemostasis was carefully achieved until there was no active bleeding.

All patients received bilateral drainage. Incise the skin with a scalpel at 1–2 cm below the incision, place two drainage tubes under the muscle layer, and fix them with 7# suture. The drainage tubes all retained three lateral holes and were placed in the space between the back of the vertebral lamina and the front of the erector spinae muscle. The outlet of the drainage tube is connected with an ordinary drainage bag (natural pressure drainage group) or a negative pressure drainage ball (negative pressure drainage group). Close the fascia, subcutaneous tissue, and skin layer by layer, and wrap them with sterile dressings.

### Application method of natural pressure drainage and negative pressure drainage device

**Fig. 1:**
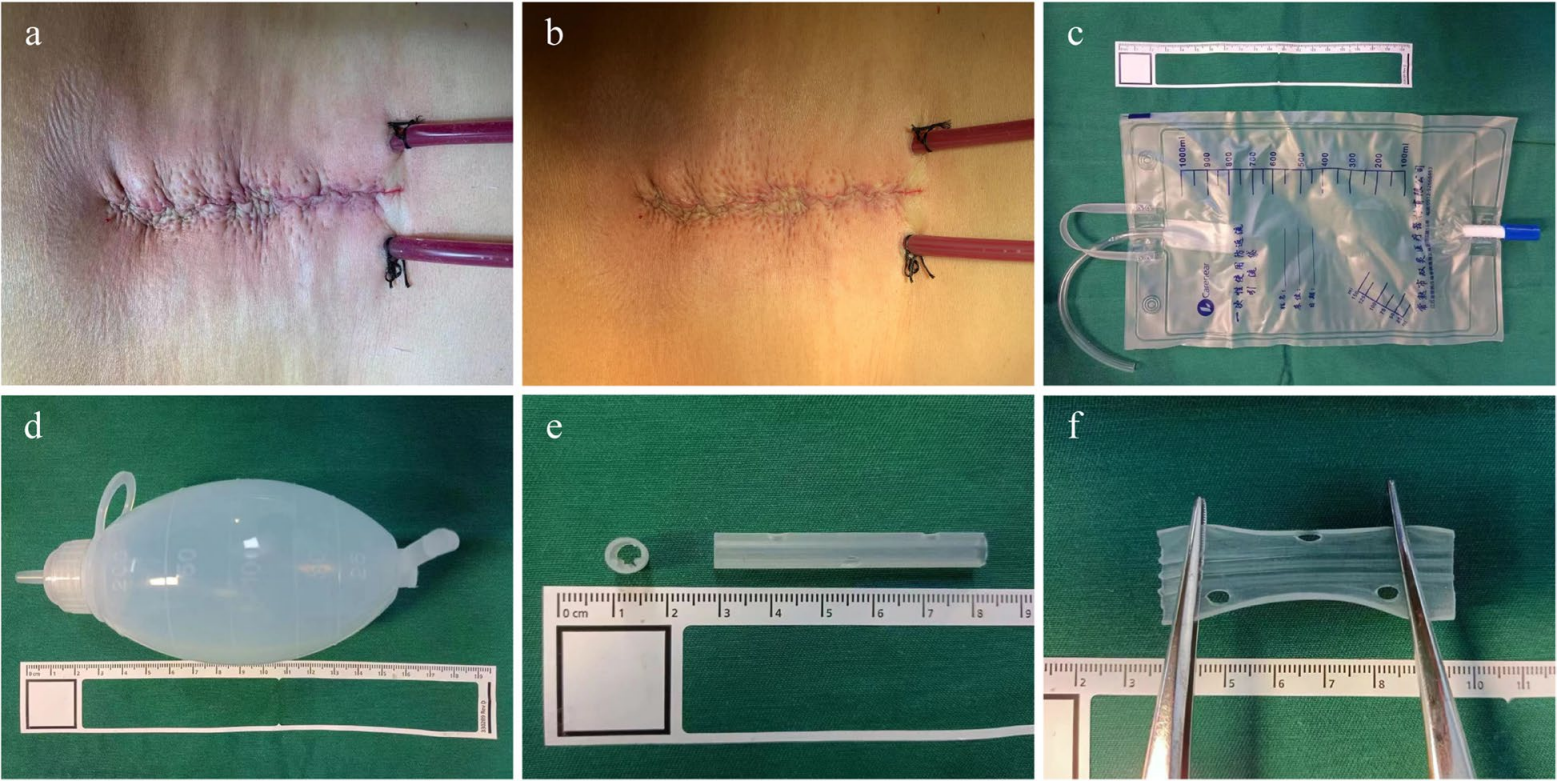
Incision appearance and drainage devices. **a**,** b** Appearance of the surgical incision with the drainage tube placed. **c** Disposable ordinary drainage bag connected with a drainage tube in natural pressure drainage group. **d** Disposable negative pressure drainage ball connected with a drainage tube in negative pressure drainage group. **e** The drainage tube used in both groups, 4.7 mm in diameter, retains three lateral holes. **f** The inner wall of the drainage tube is provided with protrusions to prevent bending and twisting

The diameter of the drainage tube is 4.7 mm. The drainage tubes of the natural pressure drainage group and the negative pressure drainage group are respectively connected with a disposable ordinary drainage bag and a disposable negative pressure drainage ball. They are all transparent and marked with scales, which are convenient for observing the drainage fluid’s properties, color, and flow. All drainage devices were placed on both sides below the level of the surgical incision to avoid backflow. The negative pressure ball always maintains a negative pressure of 10–20 kPa. The removal standard of drainage tubes is drainage volume ≤ 50ml/24 h (Fig. 1).

### Postoperative use of antibiotics, anticoagulants, and other drugs

Cefazolin sodium was used to prevent infection within 24 h after the operation, and low molecular weight heparin (4100 U, QD, 3-5 d) was subcutaneously injected 12 h after operation to prevent deep vein thrombosis. Treatments such as nerve nutrition and fluid replacement were given.

### Statistical methods

Continuous variables are expressed as (mean ± standard) deviation. Chi-squared test was used to compare categorical variables, the Shapiro-Wilk test was used to detect the normality of continuous variables, and two independent samples t-test or Mann-Whitney U test were used to compare normal or partial normal continuous variables respectively. Statistical analysis was performed using IBM SPSS Statistics 26 software, *P* < 0.05 was considered statistically significant.

## Results

There was no significant difference between the two groups in age, gender, height, weight, BMI, disease type, blood pressure on the day of surgery, preoperative albumin, hemoglobin, platelet, prothrombin time, and intraoperative bleeding (*P* > 0.05). In addition, no patients with degenerative scoliosis were included in this study because there were no patients admitted for hospitalization for degenerative scoliosis during the study period. See Table 1.

**Table 1:** General information and preoperative and intraoperative parameters of natural pressure drainage group and negative pressure drainage group

	Natural pressure drainage group	Negative pressure drainage group	*P* value
Age, year	54.72 ± 11.63	52.56 ± 10.76	0.106^a^
Gender			0.051^c^
Male	73	59	
Female	65	84	
Height, cm	167.67 ± 7.80	166.31 ± 7.43	0.102^b^
Weight, kg	67.41 ± 10.89	67.81 ± 11.15	0.431^b^
Body Mass Index (BMI)	23.88 ± 3.00	24.49 ± 3.19	0.054^b^
Disease types			0.752^c^
Spondylolisthesis	19	21	
Lumbar spinal stenosis	90	97	
Lumbar disc herniation with lumbar instability	29	25	
Degenerative scoliosis	0	0	
Blood pressure on the day of surgery, mmHg	127 ± 14 / 81 ± 10	127 ± 15 / 82 ± 10	0.994 / 0.792^b^
Preoperative albumin, g·L^− 1^	40.88 ± 3.23	41.25 ± 3.16	0.164^b^
Preoperative hemoglobin, g·L^− 1^	146.66 ± 15.93	145.10 ± 15.74	0.411^a^
Preoperative platelets, 10^9^·L^− 1^	223.88 ± 50.01	228.56 ± 52.16	0.599^b^
Preoperative prothrombin time, s	11.20 ± 0.86	11.06 ± 0.74	0.215^b^
Intraoperative blood loss, ml	386.59 ± 82.73	387.41 ± 100.60	0.861^b^

^a^By two independent samples t-test. ^b^By Mann-Whitney U test. ^c^By Chi-squared test

The albumin on the first postoperative day in the natural pressure drainage group was higher than that in the negative pressure drainage group [(33.24 ± 3.52) vs. (32.17 ± 5.03), *P* < 0.05]; The hemoglobin on the first postoperative day in the natural pressure drainage group was higher than that in the negative pressure drainage group [(126.01 ± 15.03) vs. (115.19 ± 16.25), *P* < 0.01]; The drainage volume on the first postoperative day in the natural pressure drainage group was lower than that in the negative pressure drainage group [(93.25 ± 63.58) ml vs. (119.46 ± 54.48) ml, *P <* 0.01]; The total drainage volume in the natural pressure drainage group was lower than that in the negative pressure drainage group [(355.60 ± 189.69) ml vs. (434.37 ± 149.12) ml, *P* < 0.01]; The indwelling time of drainage tube in the natural pressure drainage group was lower than that in the negative pressure drainage group [(3.29 ± 1.17) d vs. (3.45 ± 0.97) d, *P* < 0.05]. There was no significant difference in platelet count on the first postoperative day, postoperative hospital stays, and complications between the two groups (*P* > 0.05). The postoperative parameters of the two groups are shown in Table 2.

**Table 2:** Postoperative parameters of natural pressure drainage group and negative pressure drainage group

	Natural pressure drainage group	Negative pressure drainage group	*P* value
Albumin count on the first postoperative day, g·L^− 1^	33.24 ± 3.52	32.17 ± 5.03	0.035^b^
Hemoglobin on the first postoperative day, g·L^− 1^	126.01 ± 15.03	115.19 ± 16.25	0.000^a^
Platelet count on the first postoperative day, 10^9^·L^− 1^	200.47 ± 46.77	201.53 ± 52.42	0.894^b^
Drainage volume on the first postoperative day, ml	93.25 ± 63.58	119.46 ± 54.48	0.000^b^
Total drainage volume, ml	355.60 ± 189.69	434.37 ± 149.12	0.000^b^
Drainage tube indwelling time, d	3.29 ± 1.17	3.45 ± 0.97	0.023^b^
Postoperative hospital stays, d	5.17 ± 1.58	5.15 ± 1.58	0.928^b^
Complications			NA
Deep incision infection	0	0	NA
Symptomatic hematoma	0	0	NA

*NA *Not available. ^a^By two independent samples t-test. ^b^By Mann-Whitney U test

## Discussion

PLIF is a classic surgical procedure for posterior lumbar decompression, fixation, and fusion. Due to the extensive stripping of paravertebral muscles, resection of laminae, articular processes, ligamentum flavum, and other tissues, involving the handle of venous plexus in the spinal canal, the nerve roots and dural sac are directly exposed to the incision cavity, which may cause hematoma and poor drainage, resulting in serious complications such as wound infection and nerve compression [6]. Therefore, it is essential to place drainage tubes after surgery. Meanwhile, in recent years, there has also been a view of not indwelling drainage, arguing that the incidence of symptomatic hematomas and infections is not affected by whether or not they are drained [7–9]. Related studies suggest that drainage does not have a significant impact on reducing the incidence of postoperative complications or improving clinical efficacy [10], in contrast to the fact that indwelling drainage tubes requires more surgical procedures and trauma, increases blood loss and transfusion [11, 12], and can cause local discomfort, coupled with compliance problems and psychological fear, often leading to an extension of postoperative bed rest [13]. However, in clinical practice, most surgeons still routinely place drainage tubes after surgery to smoothly drain blood and exudate from surgical wounds [14], prevent the occurrence of epidural hematoma and infection [15, 16]. There is no unified standard on whether to place negative pressure drainage or natural pressure drainage in the surgical area after PLIF, as well as the specification, quantity, and placement position of drainage tubes. Ahn et al. [17] found no statistical difference between the clinical effects of drainage tubes with a diameter of 1.6 mm and 2.8 mm, so the diameter of the drainage tube had little effect on the postoperative drainage effect. According to the study by Guo et al. [18], the clinical effect of placing double drainage tubes after lumbar surgery is better than placing single drainage tube, and some studies have found that placing double drainage tubes after PLIF can prevent drainage failure caused by drainage tube blockage and other causes [19]. Therefore, it is recommended to place double drainage tubes after lumbar open surgery. Merter et al. [20] found that there was no significant difference in the cross-sectional area of the spinal cord at 24 h after surgery when the drainage tube was placed in the surgical incision, 1 cm outside the incision and 5 cm outside the incision, but the spinal cord was significantly compressed when the drainage tube was placed more than 5 cm outside the incision. Therefore, it was considered that the distance between the position of the drainage tube leading out of the skin and the surgical incision should not exceed 5 cm. In this study, natural pressure drainage and negative pressure drainage were placed 1–2 cm outside and below the bilateral incision, the diameter of the drainage tube was 4.7 mm. The clinical application effect was good, and there were no severe complications such as poor drainage and nerve compression by hematoma in the incision.

Albumin and hemoglobin are essential components in human plasma. Invisible blood loss after PLIF often leads to patients with a low nutritional status (albumin < 35 g/L), causing anemia or aggravation of anemia. Clinically, it is usually corrected by diet, the supplement of amino acid preparation and iron, and intravenous infusion of serum albumin and plasma. The results of this study show that under the same drainage tube removal standard, natural pressure drainage can reduce the loss of the above two proteins, which has the advantages of improving perioperative safety, improving postoperative symptoms such as fatigue and hypotension, promoting postoperative functional recovery, shortening hospital stay, reducing patient costs, improving patient satisfaction and saving albumin and blood resources. The drainage tube placement can affect postoperative recovery [21–23]. Patients who use natural pressure drainage can remove the drainage tube earlier and reduce postoperative pain caused by the stimulation of the drainage tube. It is in line with the concept of enhanced recovery after surgery (ERAS) [24], which is conducive to early out-of-bed activities and early intervention of rehabilitation training to improve the early function of patients and restore the self-care ability of daily life as soon as possible. In addition, the postoperative loss of albumin and hemoglobin and long-term drainage are the risk factors for complications such as surgical site infection [25–28], hemorrhagic anemia [7, 29, 30], and delirium [31, 32] after PLIF. Compared with negative pressure drainage, natural pressure drainage can reduce the level of the above risk factors and is theoretically safer.

However, this study also has some limitations. For example, none of the participants included in this study had postoperative complications such as incision infection and symptomatic hematoma, which may be related to the fact that the operation segment was a single segment and the operation time was short. The study excluded patients with diabetes, severe cardiovascular and cerebrovascular diseases, and cases with intraoperative dural rupture or postoperative cerebrospinal fluid leakage, thus not reaching the severity of infection caused by bacterial colonization [33]. Considering that the purpose of this study is to exclude bias factors, it is only relevant to the clinical efficacy of different drainage methods after PLIF. Therefore, this study needs further improvement with a multi-center, large sample, and prospective study.

To sum up, using bilateral natural pressure drainage after PLIF can reduce postoperative drainage volume, reduce postoperative albumin and hemoglobin loss, and shorten the indwelling time of the drainage tube, which can promote early ambulation of patients and facilitate early recovery. This is a safe and effective drainage method, which is more worthy of clinical application.

## Data Availability

The datasets used and analyzed during the current study are available from the corresponding author on reasonable request.

## Abbreviations

PLIF: Posterior lumbar interbody fusion
BMI: Body mass index

## References

[1] Kotil K (2016). Closed drainage versus Non-Drainage for single-level lumbar disc surgery: relationship between Epidural Hematoma and Fibrosis. Asian Spine J.

[2] Hao Q-Y, Liu C-Y, Fu C-J, Zhang X-H, Tan M-S (2016). Improved intermittent-clamped drainage in lower lumbar internal fixation. Chin Med J.

[3] Liu J-M, Deng H-L, Zhou Y, Chen X-Y, Yang D, Duan M-S (2017). Incidence and risk factors for symptomatic spinal epidural haematoma following lumbar spinal surgery. Int Orthop.

[4] Shin HK, Choi I, Roh SW, Rhim SC, Jeon SR (2017). Relevance of postoperative magnetic resonance images in evaluating Epidural Hematoma after thoracic fixation surgery. World Neurosurg.

[5] Pannell WC, Savin DD, Scott TP, Wang JC, Daubs MD (2015). Trends in the surgical treatment of lumbar spine disease in the United States. Spine J.

[6] Zeng X-J, Wang W, Zhao Z, Li M (2017). Causes and preventive measures of symptomatic spinal epidural haematoma after spinal surgery. Int Orthop.

[7] Kanayama M, Oha F, Togawa D, Shigenobu K, Hashimoto T (2010). Is Closed-suction Drainage Necessary for Single-level Lumbar Decompression?: Review of 560 Cases. Clin Orthop Related Res..

[8] Davidoff CL, Rogers JM, Simons M, Davidson AS (2018). A systematic review and meta-analysis of wound drains in non-instrumented lumbar decompression surgery. J Clin Neurosci.

[9] Zijlmans JL, Buis DR, Verbaan D, Vandertop WP (2016). Wound drains in non-complex lumbar surgery. Bone & Joint J..

[10] Guo H, Wang B, Ji Z, Gao X, Zhang Y, Yuan L et al. Closed drainage versus non-drainage for single-level lumbar discectomy: a prospective randomized controlled study. BMC Musculoskelet Disord. 2020;21(1):484. 10.1186/s12891-020-03504-x PMC7376945 32698855

[11] Liu J-M, Chen W-Z, Fu B-Q, Chen J-W, Liu Z-L, Huang S-H (2016). The Use of Closed Suction drainage in lumbar spinal surgery: is it really necessary?. World Neurosurg.

[12] Gubin AV, Prudnikova OG, Subramanyam KN, Burtsev AV, Khomchenkov MV, Mundargi AV (2019). Role of closed drain after multi-level posterior spinal surgery in adults: a randomised open-label superiority trial. European spine journal: official publication of the european spine Society, the european spinal deformity Society, and the european section of the cervical. Spine Res Soc.

[13] Adogwa O, Khalid SI, Elsamadicy AA, Voung VD, Lilly DT, Desai SA (2018). The use of subfascial drains after multi-level anterior cervical discectomy and fusion: does the data support its use?. J spine Surg (Hong Kong).

[14] von Eckardstein KL, Dohmes JE, Rohde V (2016). Use of closed suction devices and other drains in spinal surgery: results of an online, Germany-wide questionnaire. European spine journal: official publication of the european spine Society, the european spinal deformity Society, and the european section of the cervical. Spine Res Soc.

[15] Herrick DB, Tanenbaum JE, Mankarious M, Vallabh S, Fleischman E, Kurra S (2018). The relationship between surgical site drains and reoperation for wound-related complications following posterior cervical spine surgery: a multicenter retrospective study. J Neurosurg Spine.

[16] Mirzai H, Eminoglu M, Orguc S (2006). Are drains useful for lumbar disc surgery? A prospective, randomized clinical study. J Spin Disord Tech.

[17] Ahn DK, Kim JH, Chang BK, Lee JI (2016). Can we prevent a postoperative spinal epidural hematoma by using larger Diameter Suction drains?. Clin Orthop Surg..

[18] Guo L-B, Li B-T, Jiao Y-L, Pan Y-L, Guo X-W, Zhang H-S (2017). The application of bilateral placement drainage tubes in lumbar PLIF surgery Henan. J Surg.

[19] Gu Y-C, Li Y (2020). Comparison of Clinical Effects of single- and double-tube drainage after posterior lumbar intervertebral Fusion Chinese. J Mod Operative Surg.

[20] Merter A, Shibayama M (2019). Does the drain placement technique affect the amount of postoperative spinal epidural hematoma after microendoscopic decompressive laminotomy for lumbar spinal stenosis?. J Orthop Surg.

[21] Huang Q, Yao H, Xie J, Zhang S, Xu B, Pei F (2016). Early removal of drainage tube after fast-track primary total knee arthroplasty. J Knee Surg.

[22] Hung P-I, Chang M-C, Chou P-H, Lin H-H, Wang S-T, Liu C-L (2016). Is a drain tube necessary for minimally invasive lumbar spine fusion surgery?. Eur Spine J.

[23] Sun T-S, Shen J-X, Liu Z-J, Li C-D, Hong Y, Sun C-T (2017). Expert consensus in enhanced recovery after spinal surgery in China: perioperative management. Chin J Bone Joint Surg.

[24] Elsarrag M, Soldozy S, Patel P, Norat P, Sokolowski JD, Park MS (2019). Enhanced recovery after spine surgery: a systematic review. NeuroSurg Focus.

[25] Pennington Z, Lubelski D, Molina C, Westbroek EM, Ahmed AK, Sciubba DM (2019). Prolonged post-surgical drain Retention increases risk for deep wound infection after spine surgery. World Neurosurg.

[26] Liu J-M, Deng H-L, Chen X-Y, Zhou Y, Yang D, Duan M-S (2018). Risk factors for Surgical site infection after posterior lumbar spinal surgery. Spine.

[27] Schnake KJ, Pumberger M, Rappert D, Götz A, Zolotoverkh O, Waligora R (2022). Closed-suction drainage in thoracolumbar spinal surgery-clinical routine without evidence? A systematic review. European spine journal: official publication of the european spine Society, the european spinal deformity Society, and the european section of the cervical. Spine Res Soc.

[28] Li Z, Liu P, Zhang C, Xu G, Zhang Y, Chang Y (2019). Incidence, prevalence, and analysis of risk factors for Surgical site infection after lumbar Fusion surgery: ≥2-Year Follow-Up Retrospective Study. World Neurosurg.

[29] Walid MS, Abbara M, Tolaymat A, Davis JR, Waits KD, Robinson JS (2012). The role of drains in lumbar Spine Fusion. World Neurosurg.

[30] Zhao Y, Shen C-J, Zhang X (2019). Comparison of different postoperative drainage methods in lumbar fusion surgery. J Cervicodynia Lumbodynia.

[31] Shi C, Yang C, Gao R, Yuan W (2015). Risk factors for Delirium after spinal surgery: a Meta-analysis. World Neurosurg.

[32] Zhang HJ, Ma XH, Ye JB, Liu CZ, Zhou ZY. Systematic review and meta-analysis of risk factor for postoperative delirium following spinal surgery. J Orthop Surg Res. 2020;15(1):509. 10.1186/s13018-020-02035-4 PMC7643448 33153465

[33] Felippe WAB, Werneck GL, Santoro-Lopes G. Surgical Site infection among women discharged with a drain in situ after breast Cancer surgery. World J Surg. 2007;31(12):2293–9. 10.1007/s00268-007-9248-3 17917771

